# Genetic Determinants of the Anterior Cruciate Ligament Rupture in Sport: An Up-to-Date Systematic Review

**DOI:** 10.5114/jhk/163073

**Published:** 2023-07-15

**Authors:** Zhuo Sun, Paweł Cięszczyk, Kinga Humińska-Lisowska, Monika Michałowska-Sawczyn, Shuqi Yue

**Affiliations:** 1 Faculty of Physical Education, Gdansk University of Physical Education and Sport, Gdansk, Poland.

**Keywords:** gene polymorphisms, anterior cruciate injury, single-nucleotide polymorphism (SNP)

## Abstract

Anterior cruciate ligament injuries (ACLIs) are one of the most common knee injuries in sports. Although numerous factors have been related to the risk of ACLIs, it is still unclear why some individuals are more susceptible than others due to the intricate etiology of ACLIs. Several genetic factors have been identified as contributing to ACLIs. This systematic review summarizes the current evidence regarding the genetic causes of ACLIs based on the available literature. Five electronic databases were searched from 2017 to 2022. All titles, abstracts, and full texts were reviewed in detail to determine the inclusions and exclusions. The Newcastle-Ottawa Scale was used to evaluate the risk of bias. The studies' characteristics and results are presented in both narrative and tabular formats. A total of 24 studies examined 31 genes and 62 variants associated with ACLIs in the global population. Ten studies investigated seven collagens and ten SNPs for the ACL injury. The majority of studies found no significant difference in the association of the COL1A1 rs1800012, COL5A1 rs12722, VEGFA rs1570360, IL6R rs2228145, IL6 rs1800795, IL1B rs16944 and rs1143627, however, contrary results were found when nationality and gender were considered together. Conflicting evidence was found for polymorphisms rs2010963, rs699947 of the VEGFA gene in different studies. Due to a lack of data, it was impossible to determine the relationship between the anterior cruciate ligament rupture (ACLR) and the other polymorphisms. More research is required to establish a clear relationship between the ACLR and genetic variants, particularly when gender and nationality are taken into account separately.

## Introduction

Anterior cruciate ligament (ACL) injuries are one of the most common knee injuries in sports (Gwiazdon et al., 2019), and more than 70% of ACL injuries occur in a non-contact situation (Jeong, 2021). According to a report, over 250,000 of ACL injuries occur annually in the United States, and approximately 65% of those injuries require reconstructive surgery (Baker, 1998), and a long period of rehabilitation. However, roughly 45% of athletes do not return to competition (Petushek et al., 2019). Considering the increased number, high costs, and detrimental clinical consequences, the understanding of direct causes and mechanisms is needed to decrease the risk of the ACL injury.

An ACL injury is attributed to extrinsic and intrinsic mechanisms. Extrinsic factors are those that can be adjusted to decrease the risk of ACL injury such as the playing surface and exercise intensity. However, although numerous factors have been related to the risk of ACL injury, it is still unclear why some individuals are more susceptible than others due to the intricate aetiology of ACL injuries. Female athletes, for example, have a higher risk of ACL injury than male athletes (Fatahi et al., 2019) in both contact (Montalvo et al., 2019) and non-contact (Larwa et al., 2021) situations, which could be explained by female athletes to have smaller ligament size, a narrower femoral notch, an increased posterior-inferior slope of the lateral tibia plateau, increased knee and generalized laxity, and an increased body mass index (Lin et al., 2018). According to a recent study, ACL injury has a significant hereditary component, which can reach 69% in families (Magnusson et al., 2020).

In recent years, there has been an increasing amount of evidence supporting the hypothesis that genetic sequence variants play a significant role in the ACL rupture occurrence (Daohong, 2020; Kim et al., 2021), with single nucleotide polymorphisms (SNPs) in the collagen gene already having been linked to genetic susceptibility. Additionally, despite the fact that Kaynak et al. (2017, 2018) and John et al. (2016) highlighted and summarized some DNA polymorphisms, further research is needed to prove that they are directly associated with ACL injuries. Therefore, genetic factors influencing ACL injuries in sports will be updated in this systematic review based on earlier reports.

## Methods

### Protocol

In this study, the Preferred Reporting Items for Systematic Reviews and Meta-Analyses (PRISMA) statement was used for reporting (Moher et al., 2009). This systematic review was registered in the PROSPERO database and the registration number is CRD42022368810.

### Eligibility Criteria

Studies were considered for inclusion in the systematic review if they satisfied the following criteria: a case-control, cohort, cross-section and randomized controlled experiment that investigated genetic influences on the ACL injury in humans. Studies with previous systematic reviews, animal studies, book chapters, letters, editorials, conference abstracts, and review articles were disregarded. Furthermore, studies not written in English, without full-text were eliminated. Also studies with fewer than ten participants were not taken into consideration.

### Search Strategy

The electronic databases PubMed Central, Web of Science, Cochran Library, Embase and Scopus were searched from the 1^st^ of January, 2017 to the 18^th^ of September, 2022 without language restriction, however, only articles in English were taken into consideration. The following search strategy was applied: ('anterior cruciate ligament injury'/exp OR 'anterior cruciate ligament injur*':ti,ab,kw OR 'acl injur*':ti,ab,kw OR 'anterior cruciate ligament tear*':ti,ab,kw OR 'acl tear*':ti,ab,kw OR 'anterior cruciate ligament rupture*':ti,ab,kw OR 'acl rupture*':ti,ab,kw) AND ('heredity'/exp OR 'genetic determinism':ti,ab,kw OR 'genetic effect':ti,ab,kw OR 'genetic factor':ti,ab,kw OR 'genetic phenomena':ti,ab,kw OR 'genetic processes':ti,ab,kw) AND (2018:py OR 20 1 9:py OR 2020:py OR 2021:py OR 2022:py).

### Selection Process

Using the aforementioned search strategy, results were searched by the first author, and all of the results were imported into a reference manager (Endnote Vision X9.3.3) to remove duplicates. Two authors independently reviewed the results. Titles and abstracts were used as the initial criteria for selecting suitable studies. If an abstract did not provide sufficient information, full-texts were examined. In the event of discrepancies between two authors, the final decision was made by a third reviewer.

### Data Management

#### Data Items

Data from the included studies were extracted independently by two reviewers. Extracted data items included the author's name, the year of publication, the country or the region, ethnicity of research, the study design, the gene's name, a detailed genotype frequency of cases and controls, authors' definitions of cases and controls, sample size (case and control), sample types, training background of research subjects.

To evaluate the potential risk of bias in case-control, cohort and cross-section studies, the Newcastle-Ottawa Scale (NOS) (Stang, 2010) was used and scored by two reviewers independently. This scale comprises eight questions divided into three categories: research group selection, group comparability, and the ascertainment of either the exposure or the outcome of interest for case-control or cohort studies, respectively. The NOS employs a “star” rating system with a scale from zero to nine stars, and studies with an overall score of 7 were generally considered to have high quality. Each item in the selection and exposure receives one star, with a maximum of two stars provided if it meets the criteria in the comparability section. When there was a disagreement between two reviewers regarding the quality assessment of a study, a third reviewer was invited to participate in the evaluation process.

#### Data Synthesis

After analyzing the included studies, it was determined that meta-analysis was not appropriate due to the diversity of genetic variations and the heterogeneity of the risk of bias between investigations. The same genetic variants from multiple studies were compared and evaluated to determine whether genetic variables contributed to the ACLR. As a result, the findings are described in a narrative but systematic review.

## Results

### Study Selection

**Figure 1:**
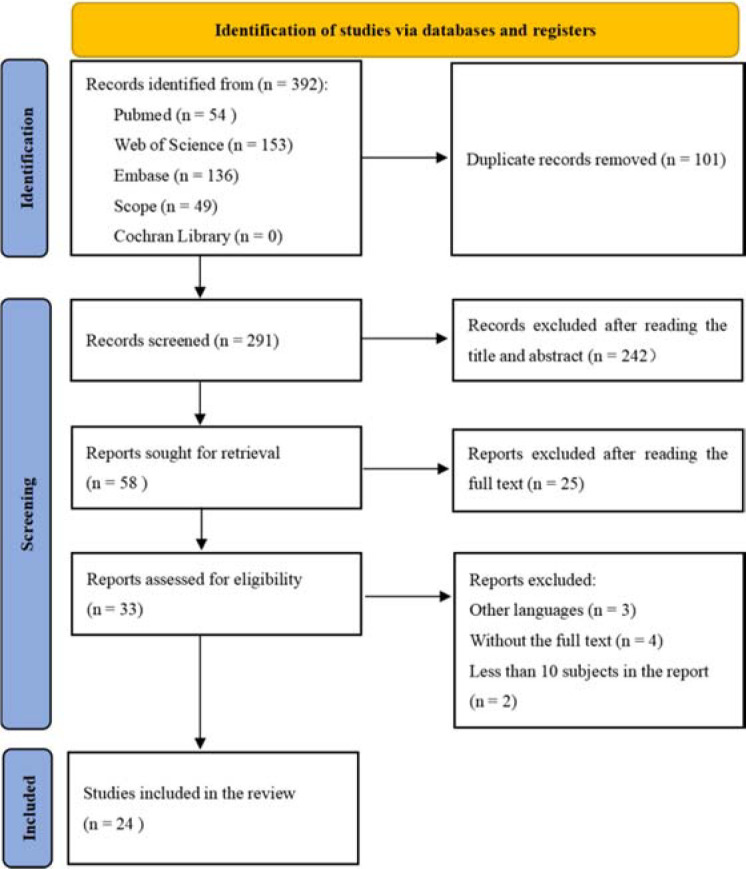
PRISMA flowchart showing the study-selection process.

From all databases, a total of 392 studies were retrieved. Two authors independently assessed the titles and abstracts of 291 papers after 101 duplicates were eliminated. Next, 242 and then 25 records were excluded after reading the title/abstract and the full text, respectively. Finally, 24 full text studies that met the criteria for inclusion were examined (Figure 1).

### Study Characteristics

This systematic review included twenty-four papers published between 2017 and 2022 in English, with summary features presented in Table 1. The total number of participants in the case and control groups was 5377 and 4343, respectively, and they could be classified into Asians, Caucasians, and colored people. Twenty-one case-control studies, two cross-sectional studies, and one cohort study were included. In the Feldmann et al.'s (2022) study, three cohorts were considered: Sweden, Poland, and Australia, while in the Suijkerbuijk et al.'s (2019) study, two cohorts were examined: Sweden and South Africa (European Caucasian ancestry). Additionally, within the studies included in the analysis, there are several independent studies conducted in South Africa (n = 9), Poland (n = 7), China (n = 1), Thailand (n = 1), India (n = 1), Brazil (n = 1), Norway (n = 1), and Sweden (n = 1). In total, thirty-one genes and sixty-two genetic variations have been reported.

**Table 1:** Characteristics of the included studies.

Study, Year	Gene		Variant	Association?	OR (95% CI)	*p* value
Wanvisa et al., 2019	Adiponectin		rs1501299 G/T	Over-representation of the GG genotype and G allele in the injury group	1.91(1.04–3.53) 1.89(1.19–3.01)	0.026 0.004
Lulińska-Kuklik et al., 2019	MMP3 MMP8 TIMP2		rs591058 C/T rs679620 G/A rs11225395C/T rs4789932 G/A	Over-represented rs679620 G and rs591058C alleles of MMP in the injury group	1.38(1.05–1.81)	0.021
Lulińska-Kuklik et al., 2020	MMP1 MMP10 MMP12		rs1799750 -/G rs486055 C/T rs2276109 T/C	No association		
Gibbon et al., 2017	MMP3		rs679620 A/G rs591058 T/C rs650108 G/A rs3025058 C/G	No association		
Lulińska-Kuklik et al., 2018	COL5A1		rs12722 C/T rs13946 C/T	Under-representation of the CT genotype of rs13946 in the injury group	Not shown	0.039
Shukla et al., 2020	COL1A1		rs1800012 G /T	No association		
Zhao et al., 2020	COL1A1 COL5A1 COL12A1 B-fibrinogen		rs1800012 G/T rs12722 C/T rs13946 C/T rs970547 A/G rs240736 C/T rs1800787 C/T rs1800788 C/T rs1800789 A/G rs1800790 A/G rs1800791 A/G rs2227389 C/T	Under-representation of the TT genotype of B-fib rs1800787 in the injury group; over-representation of rs1800788 CT, rs1800790 AG, and rs2227389 CT in the injury group; over-representation of the rs970547 A allele and AA genotype in the male injury group.	For rs970547 1.80 (A) 1.61 (AA	<0.05 0.019(A) 0.026(AA)
Laguette et al., 2020	COL5A1 TGFBR3 TGFBIT		rs3922912 G/A rs4841926 C/T rs3124299 C/T rs1805113 G/A rs1805117 T/C rs1442 G/C	TGFBR3 rs1805113 AA vs. GG TGFBI rs1442 CC vs GG	0.3(0.11–0.80) 0.3(0.12–0.77)	0.017 0.013
Gibbon et al., 2020	COL1A1		rs1107946 G/T rs1800012 G/T	No association		
Perini et al., 2022	COL1A1 COL1A2		rs1107946 G/T rs412777 A/C rs42524 C/G rs2621215 G/T	COL1A2 SNPs (rs42524 CC and rs2621215 GG) were associated with an increased risk of non-contact ACL injury	5.73(1.22–26.95) 4.29(1.26–14.61)	Not shown Not shown
Sivertsen et al., 2019	COL1A1 COL3A1 COL5A1 COL12A1		rs1800012 A/C rs1107946 A/C rs1800255 A/G rs12722 C/T rs13946 C/T rs970547 C/T	No association		
Suijkerbuijk et al., 2019	COL5A1 IL1B IL6 IL6R		rs12722 C/T rs16944 C/T rs1800795 G/C rs2228145 G/C	Over-representation of the IL6R rs2228145 CC genotype in the SA-control group	Not shown	0.028
Lulińska-Kuklik et al., 2019	VEGFA		rs699947 A/C rs1570360 A/G rs2010963 C/G	Over-representation of the VEGFA rs2010963 CC genotype in the injury group	1.85(1.11–3.08)	0.047
Lulinska-Kuklik et al., 2019	IL1B IL6 IL6R		rs16944 G/A rs1143627 G/A rs1800795 C/G rs2228145 C/A	The rs1800795 IL6 gene polymorphism was associated with the ACL rupture	1.74(1.08–2.81)	0.010 Codominant 0.022 Recessive 0.004 Overdominant
Rahim et al., 2022	IL1B IL6 IL6R VEGFA KDR		rs16944 C/T rs1800795 G/C rs2228145 C/A rs699947 C/A rs1570360 G/A rs2010963 C/G rs2071559 A/G rs1870377 T/A	Over-representation of the VEGFA rs2010963 GC and CC genotype of rs699947 in the injury group	2.43(1.00–5.87) 3.35(1.17–9.62)	0.049 0.024
Rahim et al., 2022		IL1B IL6 IL6R VEGFA KDR	rs16944 C/T rs1800795 G/C rs2228145 C/A rs699947 C/A rs1570360 G/A rs2010963 C/G rs2071559 A/G rs1870377 T/A	Over-representation of the VEGFA rs2010963 GC and CC genotype of rs699947 in the injury group	2.43(1.00–5.87) 3.35(1.17–9.62)	0.049 0.024
Rahim et al., 2017		IL1B IL6 IL6R CASP8 TNF TNFRSF1B PTGER4 TGFB2	rs16944 C/T rs1800795 G/C rs2228145 G/C rs3834129 ins/del rs1045485 C/G rs1799964 C/T rs1800629 A/G rs1061622 G/T rs4495224 A/C rs7550232 A/C	Under-representation of the IL1B rs16944 TT genotype in the female control group; over-representation of the CASP8 rs3834129 ins allele in the control group.	3.06(1.09–8.64) 1.46(1.01–2.12)	0.039 0.047
Feldmann et al., 2022		VEGFA KDR	rs699947 C/A rs1570360 G/A rs2010963 G/C rs2071559 G/A rs1870377 T/A	Under-representation of the VEGFA rs2010963 GG genotype in the SWE ACL group; Under-representation of the VEGFA AAG haplotype in the combined ACL	2.8(1.45–5.41) 0.85(0.69–1.05)	0.001 0.010
Seale et al., 2020		CASP8	rs3834129 ins/Del rs1045485 G/C rs13113 T/A	No association		
Lulińska-Kuklik et al., 2019a		TNC	rs1330363 C/T rs2104772 T/A rs13321 G/C	No association		
Gibbon et al., 2018		TNC COL27A1	rs1061494 C/T rs1138545 C/T rs2104772 A/T rs1061495 C/T rs2567706 A/G rs2241671 A/G rs2567705 A/T	Under-representation of the TNC rs2104772 AA genotype in the female control group	2.3(1.1–5.5)	0.035
Willard et al., 2018		BGN DCN COL5A1	rs1126499 C/T rs1042103 G/A rs516115 C/T rs12722 C/T	Allele combinations across BGN, COL5A1 and DCN in modulating susceptibility to ACL injury		
Ciȩszczyk et al., 2017		ACAN BGN DCN VEGFA	rs1516797 G/T rs1042103 A/G rs1126499 C/T rs516115 C/T rs699947 A/C	Under-representation of the ACAN rs1516797 G/T genotype in the control group; under-representation of the BGN rs1042103 A allele in the male control group	1.68(1.09–2.57) 1.5(1.05–2.15)	0.017 0.029

### Risk of Bias

The NOS was utilized to assess the study's quality, and the rating greater than six indicated exceptional quality. Three papers received eight points, eleven articles received seven points, seven studies received 6 points, and only three studies were rated as of low quality (Table 2).

**Table 2:** Risk of Bias Assessed by the Newcastle-Ottawa Scale.

Study	Newcastle-Ottawa Scale Score				Design
	Selection	Comparability of case	Expose	Total	
Wanvisa et al., 2019	★★★★	★★	★★	8	Case-control
Lulińska-Kuklik et al., 2019d	★★★★	★	★★	7	Case-control
Lulińska-Kuklik et al., 2020	★★★★	★	★	6	Case-control
Gibbon et al., 2017	★★★★	★	★	6	Case-control
Lulińska-Kuklik et al., 2018	★★★★	★	★★	7	Case-control
Shukla et al., 2020	★★★★	★★	★★	8	Case-control
Daohong et al., 2020	★★★★	★	★	6	Cross-sectional study
Laguette et al., 2020	★★★★	★★	★	7	Case-control
Gibbon et al., 2020	★★★	★	★	5	Case-control
Perini et al., 2022	★★★★	★	★	6	Case-control
Sivertsen et al., 2019	★★	★	★	4	Cohort study
Suijkerbuijk et al., 2019	★★★★	★★	★	7	Case-control
Lulińska-Kuklik et al., 2019b	★★★★	★	★★	7	Case-control
Rahim et al., 2018	★★★★	★★	★	7	Case-control
Shukla et al., 2020	★★★★	★	★★	7	Cross-Sectional study
Lulinska-Kuklik et al., 2019c	★★★★	★	★	6	Case-control
Rahim et al., 2022	★★★★	★	★	6	Case-control
Rahim et al., 2017	★★★★	★	★	6	Case-control
Feldmann et al., 2022	★★★	★	★	5	Case-control
Seale et al., 2020	★★★★	★	★	6	Case-control
Lulińska-Kuklik et al., 2019	★★★★	★★	★	7	Case-control
Gibbon et al., 2018	★★★★	★★	★	7	Case-control
Willard et al., 2018	★★★★	★★	★	7	Case-control
Ciȩszczyk et al., 2017	★★★★	★★	★★	8	Case-control

### Influence of Genetic Factors on the ACLR

Ten studies investigated seven different collagens and ten single nucleotide polymorphisms (SNPs) with regard to the ACLR. Some studies showed the same results with no significant difference in the association of the COL1A1 rs1800012 (Gibbon et al., 2020; Manish Shukla et al., 2020; Perini et al., 2022; Sivertsen et al., 2019; Zhao et al., 2020) and rs1107946 (Gibbon et al., 2020; Perini et al., 2022) variant with the risk of ACL ruptures (*p* > 0.05). However, when individuals of European ancestry (Swedish, South African, Polish, Norwegian and Finnish; all those participants self-identified as being of white European ancestry) were combined, the rs1800012 TT genotype (TT vs. GT + GG) was significantly over-represented in the control group compared to the ACLR group (*p* = 0.040; OR = 2.8), which confirmed a strong link between rs1800012 and the ACL risk (Gibbon et al., 2020). The A allele of rs1800012 in the COL1A1 gene was more prevalent in the Norwegian than in the Finnish cohort (minor allele frequency: 0.18 vs. 0.14; *p* < 0.03) (Sivertsen et al., 2019). Perini et al. (2022) observed that the COL1A1 SNP (rs1107946, GG or TT) was a protective association with ACLR (OR = 0.25) when the three COL1A2 SNPs (rs412777, rs42524, and rs2621215) were all wildtype.

Perini et al. (2022) reported that COL1A2 rs42524 (OR = 5.73 [1.22–26.95], and rs2621215 (4.29 [1.26–14.61) SNPs contributed significantly to the ACL risk between 146 ACLR patients and 192 healthy subjects. Sivertsen et al. (2019) showed a failed association of the COL3A1rs1800255 polymorphism and ACL injury in both Norwegian and Finnish female athletes. No COL27A1 variants were significantly associated with the risk of ACLR in South African people (European Caucasian ancestry) (Gibbon et al., 2018). Thus, there are currently insufficient evidence to support such an approach.

No significant differences were found in the genotype frequencies for the COL5A1 rs12722 polymorphism (Lulinska-Kuklik et al., 2018; Sivertsen et al., 2019; Suijkerbuijk et al., 2019; Willard et al., 2018; Zhao et al., 2020) and rs3922912, rs4841926, and rs3124299 within COL5A1 (Laguette et al., 2020). However, the frequency distributions of allele combinations may pose a risk of the ACL injury. Willard et al. (2018) showed that when all participants or only female participants were analyzed, the COL5A1 (rs12722) and DCN (rs516115) allele combination associated with an increased risk of the ACL injury (*p* = 0.006). COL5A1-IL1B-IL6 allele T-C-G combination was significantly underrepresented (*p* = 0.034) in the Swedish male cohort control group (Suijkerbuijk et al., 2019). Contradictory evidence was revealed for COL5A1 rs13946 polymorphisms (Lulinska-Kuklik et al., 2018; Sivertsen et al., 2019; Zhao et al., 2020).

COL12A1 rs970547 and rs240736 may present a high risk of the ACLR in the Chinese male population (Zhao et al., 2020), but not in the European population (Sivertsen et al., 2019). The AA genotypes of COL12A1 rs970547 were at the level of 49.3% and 27.5% in the patient and control groups, respectively (*p* = 0.026), and rs240736 of COL12A1 played a significant role in the ACL injury in Chinese men (Zhao et al., 2020). Sivertsen et al. (2019) examined 851 female Norwegian and Finnish elite athletes and found no significant differences between the ACL injury and control groups for COL12A1 rs970547 genotypes. This suggests that COL12A1 rs970547 may increase the risk of the ACL injury in males only.

The evidence was insufficient to support the influence of MMP genes on the non-contact ACL rupture risk. Lulińska et al. (2020) found no significant differences between case and control groups for the polymorphism of MMP10 (C/T rs486055), MMP12 (T/C rs2276109), and MMP1 (-/G rs1799750). Similar results were reported for rs 679620 (A/G), rs591058 (T/C) and rs650108 (G/A) in a study by Gibbon et al. (2017). In the same cohort (Lulińska-Kuklik et al., 2019d; Rahim et al., 2019), the MMP3 rs679620 G and rs591058 C alleles were significantly over-represented in cases compared to controls (OR = 1.38 [1.05–1.81], *p* = 0.021), however, no association was found for MMP8 (rs11225395C/T), and TIMP2 (rs4789932 G/A) regarding the ACL injury.

Four studies (Lulinska-Kuklik et al., 2019c; Maculewicz et al., 2019; Rahim et al., 2017, 2022; Suijkerbuijk et al., 2019) indicated the lack of significant differences in the genotype and allele frequencies for IL6R rs2228145, IL6 rs1800795, IL1B rs16944 and rs1143627 when all participants (female and male) from the control and the ACL injury group were analyzed. Only the female cohort had a significantly different genotype frequency distribution for IL1B rs16944 when compared with participants in non-contact subgroups (*p* = 0.039, OR = 3.06) (Rahim et al., 2017).

Conflicting evidence was found for polymorpsims rs2010963, rs699947 of VEGFA gene in different studies. Lulińska-Kuklik et al. (2019b), Rahim et al. (2022) and Feldmann et al. (2022) found a potential correlation between the VEGFA rs2010963 (G/C) polymorphism and the ACLR risk. However, for the associations between rs699947 and the ACLR, different results were observed (Cięszczyk et al., 2017; Feldmann et al., 2022; Lulińska-Kuklik et al., 2019b; Rahim et al., 2018, 2022; Shukla et al., 2020). There were no significant differences in the genotype or allele frequency distributions for the rs1570360 of VEGFA (Feldmann et al., 2022; Lulińska-Kuklik et al., 2019b; Rahim et al., 2018, 2022). Shukla et al. (2020) observed that the ID and II genotypes, as well as the I allele (rs35569394), were associated with a 1.64-fold increased risk of the ACL rupture compared to the control group. Rahim et al. (2022) and Feldmann et al. (2022) investigated polymorphisms of the KDR gene rs2071559 A/G and rs1870377 A/T. However, their findings did not indicate that those two SNPs had an independent relationship with the ACL injury.

Two studies analyzed the relationship between the TNC gene and the ACL risk. Lulińska-Kuklik et al. (2019a) found that the genotype and allele frequencies of TNC variants (rs1330363 C/T, rs2104772 T/A, rs13321 G/C) did not differ between cases and controls. However, another study (Gibbon et al., 2018) showed that when females were examined separately, the TNC rs2104772 (A/T) variant's AA genotype was significantly associated with the ACL rupture (*p* = 0.035, OR = 2.3).

When gender was considered, conflicting results were found for the BGN rs1042103 and rs1126499 (Cięszczyk et al., 2017; Willard et al., 2018). Cięszczyk et al. (2017) observed a significant difference in the ACAN rs1516797 genotype frequencies between the control and ACLR groups (*p* = 0.041) in Polish participants. When female and male participants were analyzed together, no significant differences in the genotype and allele distributions were noted for the DCN rs516115. In the male ACLR group, the A allele of rs1042103 (OR = 1.5) was found to be significantly over-represented, however, there were no reported significant genotype differences for the ACAN rs1516797.

Two studies investigated the effects of transforming the growth factor and tumor necrosis factor Adipokine and cytokine on ACL injury. Rahim et al. (2017) did not find any significant difference between the ACL injury and healthy groups for TGFB2 (rs7550232), TNF (rs1799964, rs1800629) and TNFRSF1B (rs1061622) when all participants (males and females) were analyzed. The TGFB rs7550232 SNP appeared to influence male weight and female height, which can provide further information to describe the genetic risk to ACL injury. Laguette et al. (2020) observed a significant difference in the frequency distribution of the rs1805113 G>A genotype (*p* = 0.033) between the two groups. The GG genotype was more prevalent in the control group than in the ACL group (*p* = 0.010, OR = 0.48). However, there were no significant differences in the genotype or allele frequency for TGFBR3 rs1805117 T > C between all groups and after sex stratification.

The limited evidence was found for the lack of association between CASP8 (rs3834129 ins/Del, rs1045485, rs13113) (Rahim et al., 2017; Seale et al., 2020), B-fibrinogenrs (rs1800789, rs1800791) (Zhao et al., 2020), PTGER4 (rs4495224) (Rahim et al., 2017) and TIMP2 (rs4789932 ) (Lulinska-Kuklik et al., 2019d; Rahim et al., 2019) variants and ACL injury. In addition, there was insufficient evidence to link Adiponectin rs1501299 (OR = 1.91) and B-fibrinogenrs (1800787, rs1800788, rs1800790, rs2227389) (Zhao et al., 2020) to the ACL injury.

## Discussion

Researchers for a long time have been interested in whether genes have a significant impact on the ACL injury. Despite the fact that numerous studies have found a link between a genetic variation and the ACL injury, conflicting results have been reported regarding the connection between single nucleotide polymorphisms (SNPs) and the ACL rupture. According to our findings, some SNPs may contribute to the ACL risk, however, more research and a larger sample size are needed for to draw a firm conclusion. This systematic review included 24 carefully designed studies published in the last six years to summarize the potential genetic variants associated with the ACL injury, and new outcomes were presented based also on previous articles by John et al. (2016) and Kaynak et al. (2017).

The majority of research has concentrated on the genes that encode for collagens, matrix metalloproteinases, interleukins, and cell signaling molecules. Previous studies have also shown that sequence variants with these genes are associated with other musculoskeletal injuries, for instance, rotator cuff tearing (Kluger et al., 2017; Tashjian et al., 2021) and Achilles tendinopathy (Abrahams et al., 2013; Pinge et al., 2012). As a multifactorial disease, none of these genetic risk factors causes the ACL rupture on their own, but rather increases the risk of the ACL rupture in susceptible individuals. Especially when gender was taken into account, results could be different. Rahim et al. (2017) found that the IL1B rs16944 TT genotype frequency was over-represented in South African females (European Caucasian ancestry) from the ACL injury group, which is in accordance with the previous statement. According to Gibbon et al. (2018), the TNC rs2104772 polymorphism was also linked to the ACL injury in females. However, the genotype of COL12A1 rs970547 (Daohong, 2020) and KDR rs2071559 (Rahim et al., 2018) have been associated to the ACL injury only in men. The existing literature provides a contradictory theory for the influence of genetic factors on ACL injuries. The assumption that females appear to have a high genetic risk of the ACL injury (Larwa et al., 2021; Montalvo et al., 2019) can be explained by several factors, including differences in the anterior tibial translation (Myer et al., 2005), landing strategy (Yu et al., 2006), neuromuscular and kinematic control and anatomic differences between females and males (Lephart et al., 2002). However, in the genetic field this assumption needs to be corroborated by further research. While the majority of studies stratified participants based on similar age, BMI, and training levels, many studies failed to subgroup the genotype by gender, implying more research with a larger sample size, as well as gender-specific research, is needed to clarify the underlying causes and mechanisms of the ACL injury.

Clearly, genetic expression differences for the ACL injury exist among populations in various countries. In the Sivertsen et al.'s(2019) study, the C allele of the SNV in the COL12A1 gene differed between the Norwegian and Finnish cohorts, and the A allele of rs1800012 in the COL1A1 gene was more abundant in the Norwegian cohort than in the Finnish one. Shukla et al. (2020) discovered no positive association between two groups of Indian athletes for the same polymorphism. When European participants from Sweden, South Africa (European Caucasian ancestry), Poland, Norway, and Finland were combined, Gibbon et al. (2020) confirmed a strong link between rs 1800012 and the ACL risk. Various studies have found similar evidence for other polymorphisms. Researchers also attempted to demonstrate broad outcomes by analyzing the general population, as a result, bias was increased. However, when discussing the impact of genetic factors on ACL injuries, it is critical to consider nationality, especially in the initial stages of research. Hence, more research needs to be carried out around the world to support consistent proof for different ethnicities.

Another noteworthy finding is that studies by Gibbon et al. (2017, 2018, 2020) and Rahim et al. (2017, 2018, 2020) used the same group of South African participants for investigating several SNP with genes, as did Lulińska et al. (2019, 2020)for Polish participants. These findings indicate that there may be one more polymorphism associated with the ACL rupture among individuals tested in those studies, which was also emphasized in a systematic review by John et al. (2016). It is biased to focus solely on one gene when considering the impact of ACL injury.

The limitations of this systematic review were that we were unable to assess the database using meta-analysis due to the diversity of genetic variations and the heterogeneity of the risk of bias between investigations. Additionally, some of the included studies used the same participants for analysis of different genes and polymorphisms, which was likely to increase the risk of bias in the results. When it comes to determining the risk of the ACL rupture, genetic tests can be a valuable tool, especially when it comes to assessing athletes, to determine their risk level. However, the results of candidate gene tests should only be used as part of a multifactorial risk model. To accurately assess the risk of sports injuries, we still need to identify specific genes that increase the risk of ACL injuries and use genetic screening as a diagnostic tool.

## Conclusions

More research is needed to establish a clear link between the ACL rupture and genetic variants, particularly gender and nationality, that need to be considered separately. Furthermore, these findings should be validated in a larger sample of subjects from around the world.
